# Two cases of airport-associated falciparum malaria in Frankfurt am Main, Germany, October 2019

**DOI:** 10.2807/1560-7917.ES.2019.24.49.1900691

**Published:** 2019-12-05

**Authors:** Imke Wieters, Philip Eisermann, Frauke Borgans, Katharina Giesbrecht, Udo Goetsch, Gudrun Just-Nübling, Johanna Kessel, Simone Lieberknecht, Birgit Muntau, Dennis Tappe, Joscha Schork, Timo Wolf

**Affiliations:** 1 Infectious Diseases, Department of Internal Medicine II, University Hospital Frankfurt, Frankfurt am Main, Germany; 2 These authors contributed equally to this work and share first authorship; 3 National Reference Centre for Tropical Pathogens, Bernhard Nocht Institute for Tropical Medicine, Hamburg, Germany; 4 Municipal Health Protection Authority, Frankfurt am Main, Germany; 5 These authors contributed equally to this work and share last authorship

**Keywords:** falciparum, malaria, airport, non-endemic country

## Abstract

Two cases of presumably airport-acquired falciparum malaria were diagnosed in Frankfurt in October 2019. They were associated with occupation at the airport, and *Plasmodium falciparum* parasites from their blood showed genetically identical microsatellite and allele patterns. Both had severe malaria. It took more than a week before the diagnosis was made. If symptoms are indicative and there is a plausible exposure, malaria should be considered even if patients have not travelled to an endemic area.

Here, we report two cases of malaria that have been diagnosed outside an endemic area. Both cases occurred in conjunction with occupation at an airport, indicating that a vector-competent mosquito was imported by air traffic. The parasites found in both cases were found to have shown genetically identical microsatellite and allele patterns.

## Case 1

**Figure 1:**
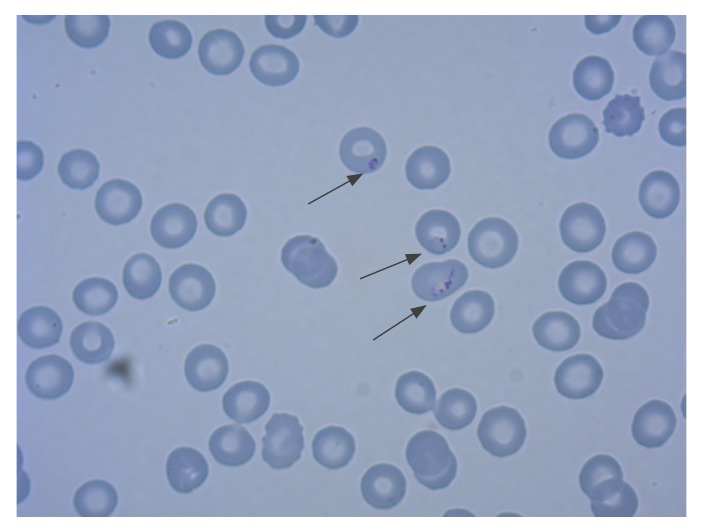
Giemsa stain of *Plasmodium falciparum* trophozoites at a parasite density of 7%, case of airport-acquired falciparum malaria, Frankfurt, Germany, October 2019 (Case 1, magnification x 1,000)

On 5 October 2019, a 38-year-old man with a positive rapid diagnostic test result for malaria was transferred to the emergency department of University Hospital Frankfurt, Germany, from a local general hospital. He had been found at home with fever, diarrhoea and confusion on the same day. The patient later reported a history of fever and malaise starting on 27 September. *Plasmodium falciparum* with a parasitaemia of 7% was demonstrated microscopically (Figure 1). Creatinine was elevated to 1.84 mg/dL (norm: max 1.2 mg/dL), but there were no other clinical signs or laboratory markers of severe malaria. He was treated with five doses of artesunate 2.4 mg/kg intravenously and received oral follow-up treatment with atovaquone/proguanil (1,000mg/400mg per day) for 3 days and recovered fully. He was discharged from hospital after 12 days and has since remained free of symptoms for 47 days.

The patient had no recent travel history, however, he had travelled to Morocco in April 2019. He did not report any underlying illness. He works at Frankfurt Airport in aircraft maintenance, and recalled that he had experienced multiple insect bites during a nightshift 2 weeks before the onset of symptoms. He had not received any blood transfusions in the past.

## Case 2

Case 2, a 51-year-old male, was transferred to the University Hospital Frankfurt on 9 October 2019 with the diagnosis of falciparium malaria and a parasitaemia of 25%. He had travelled to Algeria on 27 September, while already suffering from headache and myalgia, which he thought was an influenza-like illness. In Algeria, he visited a local doctor on 30 September when he noticed he had a fever of 38°C. Malaria was diagnosed on 6 October, 3 days after Patient 2 presented to a hospital in Algeria. He was awake but had signs of circulatory shock with a blood pressure of 60/40 mmHG and a heart rate of 115 per minute, so that he required fluid resuscitation. He was treated with quinine 16 mg/kg intravenously as loading dose and a second maintenance dose of 16 mg/kg 4 hours later. On 8 October, he was transferred by air to a local general hospital in Frankfurt/Main. Upon arrival, the patient’s condition deteriorated rapidly, reaching a Glasgow-Coma-Scale of three and he was mechanically ventilated. A cerebral computer tomography (CT) scan showed signs of cerebral swelling without necessity for neurosurgical intervention and acute kidney failure occurred (creatinine 2.16 mg/dL, norm: max 1.2mg/dL; urea 199 mg/dL, norm: max 55mg/dL). The patient was transferred to the University Hospital Frankfurt the next morning and treatment was continued with five doses of artesunate 2.4 mg/kg intravenously and oral follow-up treatment with atovaquone/proguanil (1,000mg/400mg per day) for 3 days. He made a full recovery after 16 days of inpatient treatment. Until 40 days after discharge, he remained well and free of symptoms.

Similarly to case 1, he did not have any history of recent travel to malaria endemic areas, nor had he previously had blood transfusions or underlying illnesses. He was also working at Frankfurt Airport, and on the same night shift as Case 1 but he did not recall any insect bites.

## Genotyping of *Plasmodium falciparum* from patient blood samples

An autochthonous infection with *P*. *falciparum* was suspected in both patients and whole blood samples were sent to the National Reference Centre for Tropical Pathogens at the Bernhard Nocht Institute for Tropical Medicine (BNITM) in Hamburg, Germany. In order to identify possible clonality of the two *P. falciparum* strains, we used polymerase chain reaction (PCR)-based parasite typing targeting the microsatellite PfRRM of the *rif* gene family [1], the Duffy-Binding-Like domain α (DBLα) of the *var* gene family [2] and the genes encoding for two merozoite surface proteins (MSP‐1 and MSP‐2) [3]. The markers PfRRM, DBLα and MSP-1 as well as MSP-2 contain highly polymorphic regions that can be used to differentiate between individual *P. falciparum* strains. Fragment length analysis was performed using the 3130 XL Genetic Analyzer with GeneMapper analyser software version 4.1 (Applied Biosystems, Waltham, MA, USA).

**Figure 2:**
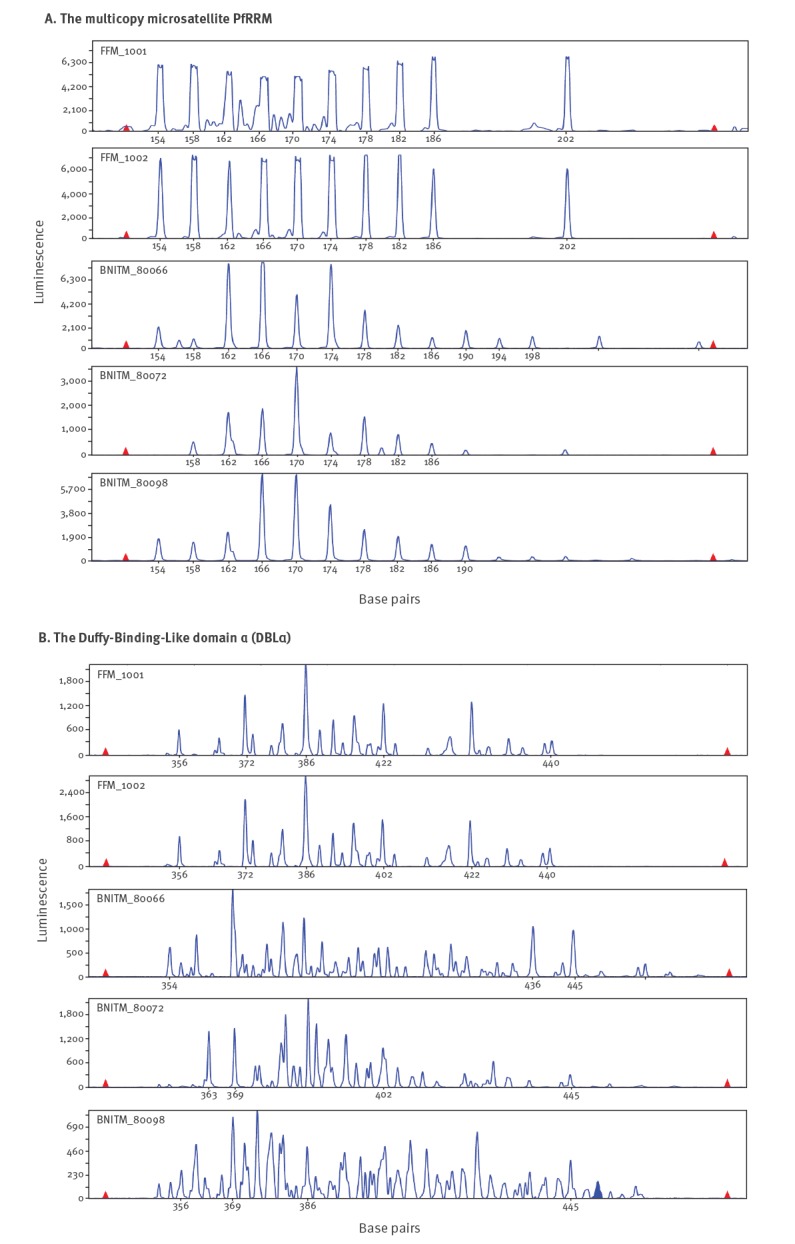
Parasite typing targeting (A) the multicopy microsatellite PfRRM and (B) the Duffy-Binding-Like domain α (DBLα) of *Plasmodium falciparum,* cases of airport-acquired malaria, Frankfurt/Main, Germany October 2019^a^

^a^ Analysis was performed on whole blood samples of both patients (FFM_1001; FFM_11002) and three samples of unrelated infections with *P. falciparum*.

Genetic typing of the two samples showed identical DNA patterns for PfRRM and DBLα respectively (Figure 2). Furthermore, MSP-1 and MSP-2 allele fragment lengths were shown to be identical as well (Table). In comparison, three randomly selected samples taken from the archive of the BNITM revealed different individual patterns (Figure 2, Table).

**Table:** MSP-1 and MSP-2 allele distribution in isolates from two cases of airport-acquired falciparum malaria^a^ compared to three unrelated *Plasmodium falciparum* infections, Frankfurt, Germany, October 2019

**MSP-1**						
	**Allelic family**	**Allele (size, bp)^b^**				
		1	2	3	4	5
**FFM_1001**	K1	**185**	n/d	n/d	n/d	n/d
**FFM_1002**		**185**	n/d	n/d	n/d	n/d
BNITM_80066		129	158	185	194	203
BNITM_80072		158	168	194	203	221
BNITM_80098		158	168	177	185	194
**FFM_1001**	MAD20	n/d	n/d	n/d	n/d	n/d
**FFM_1002**		n/d	n/d	n/d	n/d	n/d
BNITM_80066		102	110	120	167	194
BNITM_80072		131	139	n/d	n/d	n/d
BNITM_80098		102	120	158	n/d	n/d
**FFM_1001**	RO33	n/d	n/d	n/d	n/d	n/d
**FFM_1002**		n/d	n/d	n/d	n/d	n/d
BNITM_80066		125	130	n/d	n/d	n/d
BNITM_80072		125	130	n/d	n/d	n/d
BNITM_80098		125	130	n/d	n/d	n/d
**MSP-2**						
**FFM_1001**	3D7	**253**	**265**	n/d	n/d	n/d
**FFM_1002**		**253**	**265**	n/d	n/d	n/d
BNITM_80066		253	265	n/d	n/d	n/d
BNITM_80072		253	265	n/d	n/d	n/d
BNITM_80098		253	265	n/d	n/d	n/d
**FFM_1001**	FC27	**205**	n/d	n/d	n/d	n/d
**FFM_1002**		**205**	n/d	n/d	n/d	n/d
BNITM_80066		288	302	n/d	n/d	n/d
BNITM_80072		288	302	n/d	n/d	n/d
BNITM_80098		288	302	n/d	n/d	n/d

Bp: base pair; n/d: no allele detected.

^a^ FFM_1001; FFM_11002.

^b^ Different alleles within an allelic family were identified according to their size and listed numerically.

## Environmental risk assessment and vector control

On 11 October 2019, the public health authority of the city of Frankfurt contacted the employer of the patients and the airline responsible for the service hall. A list of people who worked in this aircraft service hall during the previous 4 weeks and who were on sick leave was provided; nine people were identified and contacted by phone. One of them reported mosquito bites approximately 10 days prior to the interview, but none had any symptoms of malaria.

The airline confirmed the presence of airplanes returning from three falciparum malaria endemic countries in sub-Saharan Africa in the service hall in which both patients had worked during 21 days before the symptom onset (27 September). During this period, one of them noticed mosquito bites. Considering the day temperatures of more than 20 °C during the time of likely exposure, company rooms, the aircraft service hall and the surrounding areas were inspected for possible mosquito breeding sites. No stagnant water as a possible source of reproduction of (imported) *Anopheles* mosquitoes was found. There were no further cases of malaria in the following 2 weeks in the region. The Robert Koch Institute (the national public health institute in Berlin) was asked to screen all infectious disease notifications in the Frankfurt Airport region for malaria cases without a travel history to endemic areas between 25 August and 20 October. None was reported.

## Discussion

Here we describe two cases of falciparum malaria that were presumed to have been acquired at Frankfurt Airport. While 896 imported malaria cases to Germany were reported in 2018 [4] there have been no reported, autochthonous cases. Morocco and Algeria have been declared free of malaria in 2010 and 2019 respectively [5]. In Europe, autochthonous transmissions have been described in close proximity to airports as the likely source of infection [6-15] in Belgium, France, Italy, Spain and Switzerland.

To our knowledge, there were no previously documented cases *P. falciparum* infections in employees of an airport in a non-endemic country that were found to be genetically identical. The simultaneous onset of symptoms also suggests a common source of the infections. *P. falciparum* strains in the two cases showed identical allele and microsatellite patterns using PCR-based fingerprinting. In our opinion, the autochthonous transmission of malaria parasites in central Europe remains highly unlikely. In the two severely ill cases described here, the time from symptom onset until the final diagnosis was long (9 and 12 days), a fact which has also been previously reported in airport malaria [13,14]. This circumstance demonstrates that it is crucial to consider malaria in patients who have not visited an endemic area, as a differential diagnosis if the clinical symptoms are coherent and there is a plausible mode of exposure, e.g. in the vicinity of an international airport with intercontinental flights.

Indigenous *Anopheles* species (e.g. *A. plumbeus*) showed susceptibility for *P. falciparum* in experimental infections but no natural parasite transmission has been proven so far [16,17]. In two German cases of autochthonous malaria, a local nosocomial transmission by *A. plumbeus* was suspected, however [18]. We did not have access to data concerning the mosquito monitoring for specific geographic areas in Germany nor to data for populations of indigenous or imported *Anopheles* species in the specific region around Frankfurt. The population of *A. plumbeus* in the Frankfurt area is thought to be small [19]. In the described cases, the transmission of *P. falciparum* by an infected imported mosquito seems to be highly likely. Considering sporadic reports of autochthonous malaria especially in southern Europe in the last years [20], it is important to investigate cases epidemiologically, parasitologically and entomologically, in order to distinguish so-called airport malaria as described here from transmission events originating from autochthonous mosquitoes.
